# Combining Host Genetics and Functional Analysis to Depict Inflammasome Contribution in Tuberculosis Susceptibility and Outcome in Endemic Areas

**DOI:** 10.3389/fimmu.2020.550624

**Published:** 2020-10-21

**Authors:** Dhêmerson Souza De Lima, Caio C. B. Bomfim, Vinícius N. C. Leal, Edione C. Reis, Jaíne L. S. Soares, Fernanda P. Fernandes, Eduardo P. Amaral, Flavio V. Loures, Mauricio M. Ogusku, Maria R. D'Imperio Lima, Aya Sadahiro, Alessandra Pontillo

**Affiliations:** ^1^Laboratório de Imunogenética, Departamento de Imunologia, Instituto de Ciências Biomédicas, Universidade de São Paulo, São Paulo, Brazil; ^2^Laboratório de Imunologia das Doenças Infecciosas, Departamento de Imunologia, Instituto de Ciências Biomédicas, Universidade de São Paulo, São Paulo, Brazil; ^3^Instituto de Ciência e Tecnologia, Universidade Federal de São Paulo, São José dos Campos, Brazil; ^4^Laboratório de Micobacteriologia, Instituto Nacional de Pesquisas da Amazônia, Manaus, Brazil; ^5^Departamento de Parasitologia, Universidade Federal do Amazonas, Manaus, Brazil

**Keywords:** tuberculosis, inflammasome, genetics, NLRP3, Nlrc4

## Abstract

The interplay between *M. tuberculosis* (Mtb) and humans is multifactorial. The susceptibility/resistance profile and the establishment of clinical tuberculosis (TB) still remains elusive. The gain-of-function variant rs10754558 in the *NLRP3* gene (found in 30% of the world population) confers protection against the development of TB, indicating a prominent role played by NLRP3 inflammasome against Mtb. Through genotype-guided assays and various Mtb strains (BCG, H37Rv, Beijing-1471, MP287/03), we demonstrate that Mtb strains activate inflammasome according to the NLRP3/IL-1ß or NLRC4/IL18 preferential axis. *NLRP3* and *NLRC4 g*enetic variants contribute to the presentation of TB. For the first time, we have shown that loss-of-function variants in *NLRC4* significantly contribute to the development of extra-pulmonary TB. The analysis of inflammasome activation in a cohort of TB patients and their “household contacts” (CNT) revealed that plasma IL-1ß/IFN-α ratio lets us distinguish patients from Mtb-exposed-but-healthy individuals from an endemic region. Moreover, NLRP3 inflammasome seemed “exhausted” in TB patients compared to CNT, indicating a more efficient activation of inflammasome in resistant individuals. These findings suggest that inflammasome genetics as well as virulence-dependent level of inflammasome activation contribute to the onset of a susceptible/resistant profile among Mtb-exposed individuals.

## Introduction

Tuberculosis (TB) is caused by a *Mycobacterium tuberculosis* (Mtb) infection. Only 5–10% of Mtb-infected individuals develop active TB and, in the majority of cases, the bacteria is either eliminated or remains in a latent state (1, 2). The cross-talk among host, pathogen and environment determines the outcome of an infection (3).

Host innate immune genes have been recently taken into account to explain how the first host/pathogen contact could shape one's subsequent response to Mtb (resistance vs. susceptibility). After accessing the host airways, Mtb is phagocyted by alveolar macrophages which initiate an innate immune response that leads to pathogen eradication, with the occasional contribution of an adaptive cell-mediated response (mainly driven by interferon (IFN)-γ) (4). Alternatively, a chronic inflammatory process begins with Mtb persistance in the lung and that may result in tissue damage through the formation of granulomas and lung fibrosis. In more severe infections, a dramatic lung parenchyma destruction occurs (5, 6). As such, the host innate immune response and further tissue inflammation could either be beneficial or detrimental in the fight against Mtb, possibly due to a delicate balance among several mediators.

Recently, Mayer Barber et al. showed that the plasma level of interleukin (IL)-1ß, type I IFN (IFN-I) and prostaglandin (PG)-E2—among several other inflammatory mediators—characterized the clinical presentation (severe vs. mild) of Mtb infections in TB patients (7), suggesting that a fine-tune regulation of innate immune response is crucial for TB outcomes.

Inflammasome is a cytosolic multi-protein complex that mediates the maturation and release of IL-1β and IL-18 (8). Pathogen- or damage-associated molecular patterns (PAMPs and DAMPs, respectively) activate the inflammasome through a group of cytosolic pathogen recognition receptors (PRRs), including NLRP1, NLRP3, NLRC4, NAIP, AIM2, and pyrin (8, 9). During infection, pathogens carry or inject PAMPs into cell cytosol and/or induce damage and break into the host homeostasis through the release of DAMPs. These are detected by inflammasome sensors, which in turn can recruit the adaptor molecule ASC and the effector molecule caspase-1, which is responsible for the maturation of IL-1ß and IL-18. Moreover, caspase-1 can mediate the cleavage of the pore-forming protein gasdermin D, leading to pro-inflammatory active cell death called pyroptosis (8).

Inflammasome activation constitutes a very efficient and rapid mechanism for host defense against pathogens. In this case, the inflammation is triggered either by leukocyte recruitment, *via* IL-1ß and IL-18 or by the destruction of the pathogen *niche* and inflammation amplification through pyroptosis. However, pathogen-induced response may be excessive with inflammasome-mediated tissue damage or, on the other hand, pathogens may evade host detection and/or inflammasome activation itself (9).

In murine bone marrow-derived macrophages, Mtb activates inflammasome through NLRP3 (10, 11) and mice lacking NLRP3 showed a reduced IL-1β production in response to an Mtb infection (12), suggesting that this sensor is crucial to trigger an animal response to this bacteria.

Besides being one of the most studied inflammasome sensors, researchers still have not completely demonstrated how NLRP3 is activated, as it responds to several PAMPs and DAMP, such as bacterial lipopolysaccharide (LPS), bacterial secretion systems, bacterial toxins, viral proteins, nucleic acids, increased extracellular concentration of ATP and alarmins (released by dead cells), organic and inorganic crystal or fibers (i.e., uric acid, cholesterol, ß-amyloid). Scientific evidence indicate that PAMPs and DAMPs activate NLRP3 through the induction of cytosolic alterations, such as K+ efflux, mediated by membrane pore-forming toxins or the binding of extracellular ATP to the purinergic receptor P2X7; or even in the case of damage in cell organelles (mitochondria or lysosome) with the consequent release of their contents, including reactive oxygen species, mitochondrial DNA or lysosomal cathepsins (8). As NLRP3 can be activated by such a plethora of signals, it is strongly regulated at several levels, including transcription and post-transcriptional events, including the feed-back mechanism induced by nitric oxide (NO), interferons and PGE2 (13–15).

Intriguingly, Mtb not only induces NLRP3 inflammasome activation but also the production of NO and IFN-I in mice (16). Accordingly, Wasserman et al. have shown that in THP-1, the human monocytic cell line, Mtb H37Rv triggers both NLRP3 and AIM2 inflammasome activation and IFN-1 production, most likely as an escape mechanism (17). In TB patients, the relative balance between the serum level of IL-1ß, IFN-I, and PGE-2 allows for classification according to disease severity (7). Altogether, these findings suggest that correct NLRP3 inflammasome activation can certainly contribute to bacterial clearance.

We previously reported the protective gain-of-function effect of a Single Nucleotide Variant (SNV) in the *NLRP3* 3'UTR region (rs10754558; found in about 30% of the world population) against the development of pulmonary TB (18). This led us to hypothesize genes coding factors taking part in the inflammasome assembling and function, which directly influences the IL-1ß and/or IL-18 production, could affect the outcome of an Mtb infection, therefore determining either the susceptibility and/or the prognosis for a TB patients.

In this study, we proposed a model of host/pathogen interplay including host inflammasome genetics, Mtb aggressiveness and inflammasome response rate, to evaluate the relative contribution of each parameter in individual TB susceptibility and outcome.

## Materials and Methods

### Cohort for Cellular Assays

Three groups of adult volunteers were recruited for the *in vitro* study: healthy donors (HD) from TB non-endemic area, TB patients and their household contacts from TB endemic area. Thirty unrelated healthy volunteers from the metropolitan area of São Paulo (SP, Brazil) were recruited at the Blood Bank of “Oswaldo Cruz” hospital. Twenty-seven unrelated adults recently diagnosed with active pulmonary TB were recruited at the Reference Centre for Sanitary Pneumology “Cardoso Fontes” (Manaus, AM, Brazil). Twenty-seven unrelated adults in daily contact with TB patients (“household contacts”) but without TB symptoms and negative for Mtb detection tests were included in the study as Mtb-exposed TB negative individuals. Diagnosis of active pulmonary TB is based on the evaluation of clinical symptoms together with Mtb detection tests: sputum smears analyses (19, 20) and/or GeneXpert MTB/RIF tests (C*epheid*). Main characteristics of the volunteers recruited for the study are presented in Table 1.

**Table 1:** Characteristics of the individuals recruited for the cellular assays.

**Characteristics**	**HD (*n* = 30)**	**TB (*n* = 27)**	**CNT (*n* = 27)**
State of origin	SP	AM	AM
Age, years (Mean ± SD)	45 ± 13	51 ± 11	52 ± 12
Male/Female, *n* (%)	14/16 (46.7/54.3)	16/11 (59/41)	11/16 (41/59)
BCG vaccinated, *n* (%)	30 (100)	27 (100)	27 (100)
TST+, *n* (%)	0	5 (18.6)	3 (11.1)
IGRA+, *n* (%)	n.d.	12 (44.4)	13 (48.1)
Mtb detection test +, *n* (%)	n.d.	27 (100)	0
Mtb detection test in sputum smear +, *n* (%)	n.d.	13 (50)	0

*State of origin in Brazil, mean age, gender distribution and BCG vaccination are reported for the three groups of studied individuals: healthy donors (HD; n = 30), TB patients (TB; n = 27) and contacts (CNT; n = 27). Positive results for TST test, IGRA, Mtb detection test as well as s Mtb detection test in the sputum smear were indicated for the TB patients and contacts*.

*SP, São Paulo State; AM, Amazonas State; n: number of individuals; SD, standard deviation; %, percentage; BCG, Bacillus Calmette Guerin; TST, Tuberculin Skin Test or Mantoux test; IGRA, Interferon Gamma Release Assay; Mtb, Mycobacterium tuberculosis*.

### Cohort for Genotyping Study

Genomic DNA from 352 unrelated TB patients and 288 healthy adults (without clinical signs or symptoms of TB) from the Brazilian State of Amazonas (not indigenous people from the Amazon forest) was used for the genetic association study. Diagnosis of TB is based on the evaluation of clinical symptoms together with Mtb detection tests, as above mentioned. Main characteristics of this cohort are presented in Table 2.

**Table 2:** General characteristics of the TB cohort for genotyping study.

**Characteristics**	**TB (*n* = 288)**	**ETB (*n* = 64)**	**HD (*n* = 288)**
**Gender**			
Male, *n* (%)	193 (67.0%)	30 (47.0%)	155 (46.4%)
Female, *n* (%)	95 (33.0%)	34 (53.0%)	133 (53.6%)
Mean age ± SD	38.2 ± 13.2	33.0 ± 13.02	35.8 ± 12.0
**BCG–vaccinated**			
Yes, *n* (%)	212 (73.6%)	55 (86.0%)	180 (62.5%)
No, *n* (%)	25 (8.70%)	9 (14.0%)	36 (12.5%)
Data not available, *n* (%)	51 (17.7%)		72 (25.0%)

*Mean age, gender distribution and BCG vaccination are reported for the three groups of studied individuals from the Brazilian State of Amazonas: healthy donors (HD; n = 288), pulmonary TB (TB; n = 288) and extra-pulmonary (ETB; n = 64)*.

### Mycobacterial Culture Conditions and Preparation for Cell Infection

Non-virulent Bacillus Calmette-Guerin (BCG), common virulent H37Rv and hyper-virulent Beijing 1471 (Bj) and MP287/03 (MP) mycobacterium strains were used in this study. *M. tuberculosis* H37Rv and Beijing 1471, *M. bovis strains* Bacillus Calmette-Guerin and MP287/03 were sourced from the laboratory of Professor Maria Regina D'Imperio Lima (Department of Immunology, Institute of Biomedical Sciences, University of São Paulo). Mtb were grown at 37°C, either in 7H9 medium (*Difco*) supplemented with 10% albumin-dextrose-catalase (ADC, *Middlebrook*) or on 7H10 agar plates supplemented with 10% oleic acid-albumin-dextrose-catalase (OADC, *Middlebrook*). Bacteria were grown to the exponential phase (optical density at 600 nm, OD_600_, of 0.1), washed once in a 7H9 medium, and resuspended in 7H9 to an OD_600_, of 1—equivalent to 2 x 10^8^ bacilli/ml for BCG, 1 x10^7^ bacilli/mL for H37Rv and Bj, 2 x 10^7^ for MP. The required volume for Mtb bacterial suspension was then added to the cell culture medium for infection of human monocyte-derived macrophages (MDM) at a multiplicity of infection (MOI) of 0.033. This MOI was selected after pilot experiments as the minimum MOI capable of activating IL-1ß production in MDM. We avoided using a higher MOI in an attempt to mimic natural infection in humans (21). For colony formation unit (CFU) assays, infected MDM were lysed and serial dilutions were plated in a complete agar medium. After 21 days, bacterial growth in MDM was evaluated by CFU counting.

### MDM Generation and *in-vitro* Infection

Peripheral blood mononuclear cells (PBMC) were isolated from donor blood using the Ficoll-Paque® density gradient centrifugation (*GE Healthcare, Biosciences*). Monocytes were separated from total PBMC by plastic adherence and cultivated at 0.4 × 10^6^ cells/mL in RPMI-1640 (*Gibco, ThermoFisher Scientific*), supplemented with 10% fetal bovine serum (FBS; *Gibco*) and 25 ng/mL monocyte colony stimulating factor (M-CSF; *PreproTech*) for 5 days at 37°C in 5% CO_2_ atmosphere to obtain MDM. Monocytes-to-MDM differentiation was confirmed by flow cytometry analyses of the CD14 and CD68 surface markers (Supplementary File 1). MDM were infected with H37Rv, Bj, MP, and BCG strains at MOI 0.033 for 3 h, washed, and then cultivated for 24 h. In some experiments. MDM were treated with 10 μM parthenolide (PTD; *Sigma-Adrich*), a general inhibitor of inflamamsome (22); or 10 μM MCC-950 (*InvivoGen*), a specific inhibitor of NLRP3 (23); or 10 μM CA074-Me (CA074; *Merck*), an inhibitor of intracellular cathepsin B; or 10 μM of cytocalasin-D (Cyt-D; *Sigma-Aldrich*), an inhibitor of actin polymerization which impairs phagocytosis. PTD, MCC-950, and Ca074 were added to MDM cultures for 1 h prior to bacterial infection. Cyt-D was added to MDM cultures during *in vitro* infection. In some experiments, 1 mM ATP (*Sigma-Aldrich*) was added at the end of treatment for another 15 min. The treatment with 1 μg/mL LPS (*E. coli* strain O111:B4; *Sigma-Aldrich*) for 24 h and 1 mM ATP for another 15 min were used as positive control for NLRP3 inflammasome activation (24, 25). For CFU assays, 10 ng/mL of recombinant human IL-1ß (rh-IL-1ß; *Peptotech*) or 10 μM PTD were added to the culture. Inflammasome activation was analyzed by IL-1ß and IL-18 release in the culture supernatants, ‘cleaved/activated caspase-1 and “speck” formation.

### Cytokines Quantification

IL-1ß, IL-18, IFN-ß, and TNF levels in serum and culture supernatants were measured using commercial ELISA Kits (*Biolegend, R&D Systems*) according to the manufacturer's instructions.

### Caspase-1 Activity Assay

The detection of caspase-1 activity in MDM FAM-FLICA® Caspase-1 Assay Kit (*Immunochemistry Technologies*) and flow cytometry, according to the manufacturer's instructions. Briefly, 2 x 10^5^ MDM were stimulated according to the above-mentioned protocol, and then incubated with the fluorescent inhibitor probe FAM-YVAD-FMK for 1 h at 37°C, 5% CO_2_. The samples were then washed, incubated with Live/Dead Fixable Cell Stain Kit (*Thermo Fisher Scientific*), and analyzed by flow cytometry. The caspase-1 activity was expressed as percentage of FAM-FLICA positive (+) live cells.

### Cell Cytotoxicity

Cell cytotoxicity was assessed by the quantification of Lactate dehydrogenase (LDH) release in supernatants using the LDH Cytotoxicity Assay Kit (*ThermoFisher Scientific*), according to the manufacturer's protocol. The cytotoxicity is expressed as percentage of LDH release relative to the positive control (Triton).

### “Specks” Formation

Inflammasome complex assembly was evaluated by the detection of “specks" formation using immunofluorescence microscopy. MDM were fixed and permeabilized with Cytofix/Cytoperm reagent (*BD Biosciences*) for 30 min at 37°C and 5% CO_2_ and incubated with primary antibody for NLRP3 (1:100 mouse anti-human NLRP3, *Abram*) and/or NLRC4 (1:200 rabbit anti-human NLRC4; *Biolegend*) overnight at room temperature. Fluorescent secondary antibodies (Alexa 488-conjugated goat-anti-mouse IgG1, or Alexa 647-conjugated goat-anti-rabbit IgG1; *Thermofisher Scientific)* were then added for 1 h. 4',6-Diamidine-2'-phenylindole dihydrochloride (DAPI; *Sigma-Aldrich*) was used for nuclear counterstaining. Image acquisition was performed at the microscope facility at the Laboratory of Cellular Biology from the Butantan Institute (São Paulo, SP, Brazil) using a DMi8 confocal laser scanning microscope equipped with a digital camera DFC310 FX (Leica). The counting of NLRP3+ and NLRC4+ *specks* in MDM was performed manually by observing speck formation within the cells in 10 fields (26).

### Inflammasome' Genes Expression Analysis

Total RNA was isolated from 0.4 × 10^6^ MDM using the RNAqueous-Micro kit (*Ambion, Thermo Fisher Scientific*), according to the manufacturer's protocol, and quantified using Nanodrop N-1000 (*Agilent*). Total RNA was converted into cDNA using Superscript III RT kit and random primers (I*nvitrogen, Thermo Fisher Scientific*). *NLRP3* (*Hs00366465*), *NLRC4* (*Hs00368367*), *IL1B* (hs01555410), and *IL18 (Hs01038788*) genes were amplified using TaqMan® gene-specific assays (*Applied Biosystems, Thermo Fisher Scientific*) and qPCR on the QuantStudio 3.0 Real-Time PCR equipment (*Applied Biosystems*). The QuantStudio 3.0 software was used to obtain cycle threshold values (Ct) for relative gene expression analysis according to the Fold Change (FC) method (27). Raw expression data (Ct) were normalized with the expression of the house-keeping gene glyceraldehyde-3-phosphate dehydrogenase/*GAPDH* (*Hs02758991; TaqMan*^®^ *assay*) (ΔCt), and the FC was calculated comparing stimulated and unstimulated (UN) conditions (FC=2^−−ΔΔCt^; ΔΔ*Ct* = Δ*Ct*_stimulated_−Δ*Ct*_UN_). Alternatively, basal (constitutive) gene expression was calculated as 2^−ΔCt^.

### SNVs Genotyping

*NLRP3* rs10754558 C>G and *NLRC4* rs479333 G>C single nucleotide variants were selected based on their known functional effect, and elevated minor allele frequency (MAF) in the general population (0.30 and 0.49 respectively, from *www.ensembl.org*). *NLRP3* rs10754558 is a 3'UTR variant previously associated with increased *NLRP3* mRNA stability and supposed augmented inflammasome activation (28). *NLRC4* rs479333 is an intronic polymorphism which negatively affects *NLRC4* expression (29) and serum IL-18 level (30). They were genotyped in the TB cohort with TaqMan®-type allele-specific commercial assays (*Applied Biosystems*) and qPCR on the QuantStudio 3.0 Real-Time PCR equipment. The QuantStudio 3.0 software was used for allelic discrimination.

### Data Analysis

All data were collected and analyzed from at least three independent experiments. A normality test was applied to the data, and a parametric or non-parametric analysis was used accordingly to compare two or more data sets, as specified for each graph. The level of significance was *p* < 0.05. Calculations were performed using the statistical software package GraphPad Prism (v8.01). Genotyping data were analyzed through multivariate analysis according to the general linear model (GLM) using the package SNPassoc and R-project. Bonferroni correction for multiple comparisons (2 SNVs) required a *p* < 0.025 (0.05/2).

## Results

### *M. tuberculosis* Induces Inflammasome Activation and IL-1ß Production Plays a Major Role in Bacterial Restriction *in vitro* as Well as *in vivo*

Mtb triggers NLRP3 inflammasome in mice (10–12), as well as in some *in-vitro* models of human macrophages (17, 31, 32); however, the multiplicity of infection (MOI) implied is always high (1–10 or more) compared to what expected during natural infection (<<1) (21).

Considering this, we first investigated whether the common virulent reference strain Mtb H37Rv activates the inflammasome at a physiologic MOI. For this, we measured cytokine production and caspase-1 activation at a MOI of 0.033 in Mtb-infected monocyte-derived macrophages (MDM).

**Figure 1:**
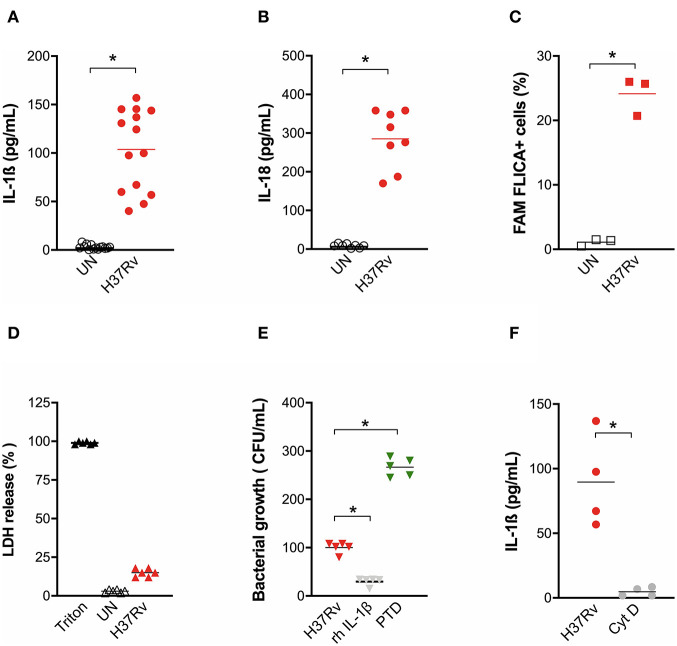
*M. tuberculosis* strain H37Rv induces inflammasome activation in human MDM and IL-1ß production plays a major role in bacterial restriction. The healthy donors' monocyte-derived macrophages (MDM; *n* = 14) were challenged with the *M. tuberculosis* (Mtb) strain H37Rv (MOI: 0.033) for 3 h, then washed and cultured for 24 h. The release of IL-1ß (*n* = 14) **(A)** and IL-18 (*n* = 8) **(B)** was measured in culture supernatants of untreated (UN) and infected (H37Rv) MDM. Cells were harvested for an analysis of caspase-1 activity by a FAM-FLICA® assay and flow cytometry from three independent experiments. The percentage of FAM-FLICA+ cells was reported for H37Rv-infected (H37Rv) and untreated (UN) MDM **(C)**. Lactate Dehydrogenase (LDH) liberation was measured in culture supernatants in three independent experiments. Data are expressed as a percentage in respect to the positive control (Triton, 100% cell lysis) **(D)**. The healthy donors' MDM (*n* = 5) were infected with Mtb H37Rv (MOI: 0.033) for 3 h in the presence of exogenous recombinant human IL-1ß (rh-IL-1ß) or parthenolide (PTD; 10 μM), then lysed. Serial dilutions were plated in a complete agar medium. Bacterial growth was evaluated after 21 days by counting colony formation units (CFU) **(E)**. The healthy donors' MDM (*n* = 4) were infected with Mtb H37Rv (MOI: 0.033) for 3 h in the presence of cytochalasin D (Cyt D; 10 μM), then washed and cultured for 24 h. The release of IL-1ß was measured at the end of incubation in infected MDM (H37Rv) and infected MDM in the presence of a phagocytosis inhibitor (Cyt D) **(F)**. Individual data are reported with their mean. The Mann–Whitney test was used to compare treated and untreated groups **(A–C,F)**. The Kruskall–Wallis test followed by a Bonferroni post-test were used to compare H37Rv, rh-IL-1ß, and PTD groups **(E)**. Differences with a *p* < 0.05 were considered statistically significant (*).

At this MOI, H37Rv was also able to induce a significant release of inflammasome cytokines IL-1ß (Figure 1A) and IL-18 (Figure 1B) from MDM, which was accompanied by the increase of caspase-1 cleavage (Figure 1C) but not by a significant release of LDH (Figure 1D), suggesting the induction of the canonical pathway of inflammasome activation, but without high rates of pyroptosis.

Little is known about a possible function of IL-18 against mycobacteria, while IL-1ß plays a role in macrophages phagocytosis (33) Therefore, we performed a bacterial growth assay in Mtb-infected MDM in the presence of exogenous recombinant IL-1ß or pre-treated with the inflammasome inhibitor parthenolide (PTD). H37Rv bacterial growth was significantly inhibited by IL-1ß (70.2% CFU inhibition). At the same time, a significant increase of Mtb growth was observed when PTD was added to cultures (160% CFU increase) (Figure 1E). H37Rv-driven inflammasome activation seems to be direct, as the inhibition of mycobacterial phagocytosis completely abolished IL-1ß production (Figure 1F).

These data emphasize that the activation of inflammasome and IL-1β production are mandatory for Mtb containment.

### NLRP3 Contributes to Inflammasome Activation by the *Mtb* Common Virulence Strain H37Rv in Human MDM

**Figure 2:**
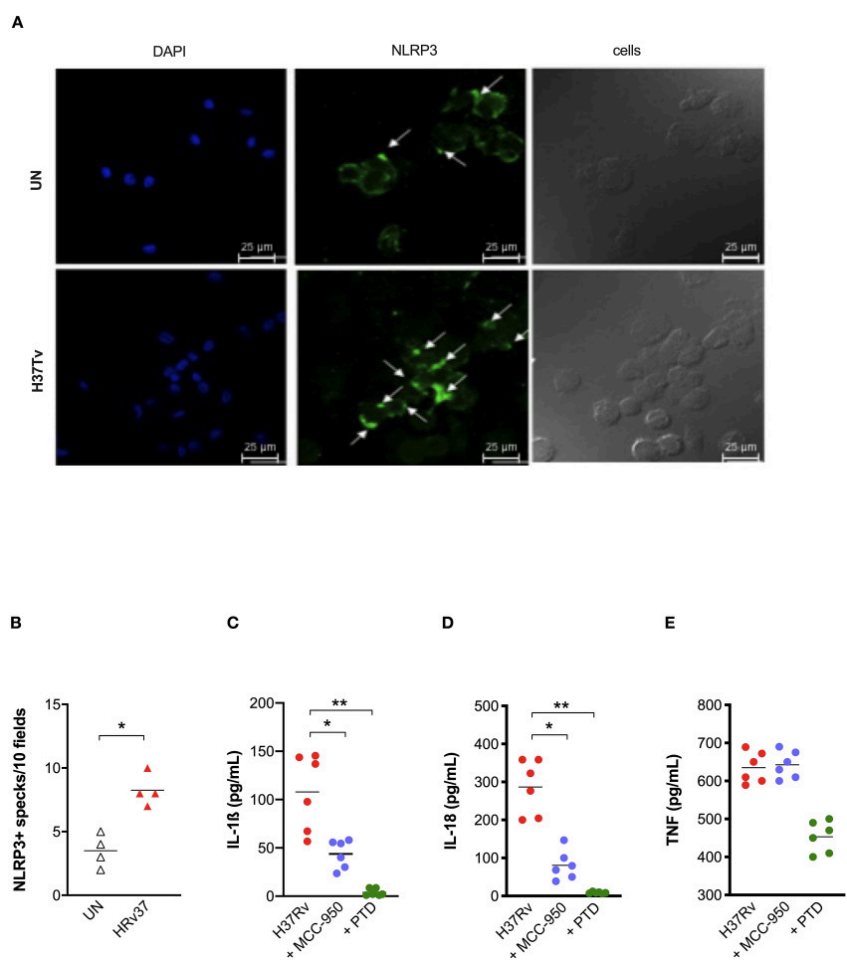
NLRP3 contributes to inflammasome activation by *Mtb* H37Rv in human MDM. The healthy donors' monocyte-derived macrophages (MDM; *n* = 4) were cultivated (0.08 × 10^6^) in 16-well chamber slides (*Thermo Fisher Scientific*), challenged with *M. tuberculosis* (Mtb) H37Rv (MOI: 0.033) for 3 h, then washed and cultured for 24 h. Mouse anti-human NLRP3 and a fluorescent secondary antibody (Alexa 488-conjugated goat-anti-mouse IgG1) were used to label NLRP3. DAPI was used to counterstain the nuclei. NLRP3+ speck formation in untreated (UN) and infected MDM (H37Rv) was analyzed using fluorescence microscopy. A representative experiment was reported. Arrows indicated NLRP3+ specks. **(A)**. The number of NLRP3+ specks was manually counted for each independent experiment **(B)**. The healthy donors' monocyte-derived macrophages (MDM; *n* = 6) were treated with 10 μM MCC-950 or 10 μM parthenolide (PTD) 1 h before infection with *M. tuberculosis* H37Rv (MOI: 0.033) for 3 h, then washed and cultured for 24 h. The release of IL-1ß **(C)**, IL-18 **(D)**, and TNF **(E)** was measured in culture supernatants. Individual data are reported with their mean. The Mann–Whitney test was used to compare treated and untreated groups **(B)**. The Kruskall–Wallis test followed by a Bonferroni post-test were used to compare H37Rv, MCC-950 and PTD groups **(C–E)**. Differences with a *p* < 0.05 were considered statistically significant (*). **p < 0.001.

As our previous results pointed out the protective role of a gain-of-function genetic variant in *NLRP3* (rs10754558) (18), and given the central role of this receptor in the mouse model of Mtb infection as well (10–12), we next asked whether the observed activation of inflammasome complex by Mtb H37Rv in MDM could be dependent on NLRP3. The formation of *specks* indicates the activation of inflammasome and also allows for the visualization of a mounted complex platform, including the activated sensor (26). Thus, we subsequently replicated MDM infection with Mtb H37Rv to confirm NLRP3 participation in inflammasome responses. To do so, cells were stained using specific antibodies and samples analyzed at the end of infection assay under a fluorescence microscope. A significant increase of NLRP3+ specks were detected in Mtb-infected MDM compared to untreated cells (Figures 2A,B).

Next, we applied another approach, based on the use of known chemical inhibitors, to confirm NLRP3 involvement in the response. IL-1ß and IL-18 release was measured in Mtb infected-MDM and pre-treated with the NLRP3 inhibitor MCC-950 (10 μM) (23). As a positive control of inhibition, we also pre-treated MDM with the general inflammasome inhibitor parthenolide (PTD; 10 μM) (22). As expected, PTD entirely abolished cytokines production, while MCC-950 significantly reduced, but not completely abrogated, the release of IL-1ß (Figure 2C) and IL-18 (Figure 2D) in infected-MDM (48.7 and 71.7% inhibition, respectively). The secretion of a non-inflammasome-dependent pro-inflammatory cytokine, TNF, was not significantly affected by the use of inhibitors (Figure 2E).

Altogether, these findings corroborate previous data about the importance of NLRP3 in macrophage' response against virulent Mtb (10–12, 17, 31, 32), including at physiologic condition of infection (MOI: 0.033), and support the protective effect observed for *NLRP3* rs10754558 against the development of active pulmonary TB (18).

### TB Patients Present an Impaired NLRP3 Response and IL-1ß Release

**Figure 3:**
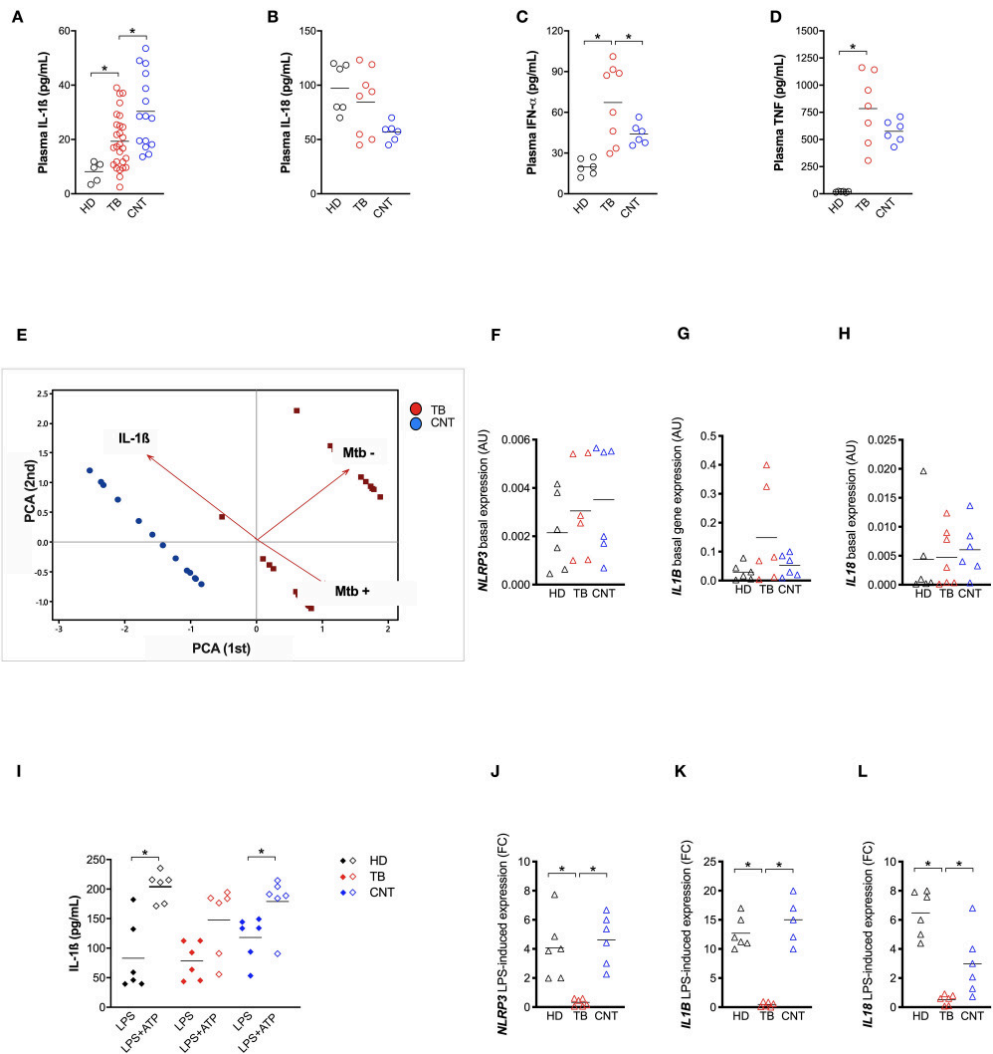
TB patients present an impaired NLRP3 inflammasome response. IL-1ß **(A)** level were measured in plasma from recently diagnosed untreated TB patients (TB; *n* = 27) and some of their “household contacts” (CNT; *n* = 15) from an endemic TB area (Manaus, metropolitan region, AM) and from healthy donors in a non-endemic area (São Paulo metropolitan region, SP) (HD; *n* = 5). IL-18 **(B)**, IFN-a **(C)**, and TNF **(D)** dosage was executed in a group of samples (HD, *n* = 6; TB, *n* = 8; CNT, *n* = 6). **(E)** A Principal Components (PC) analysis was executed including the plasma level of IL-1ß, a mycobacteria count in a sputum analysis and a diagnosis of TB (TB or CNT). Monocyte-derived macrophages (MDM) were isolated from HD (*n* = 6), TB patients (*n* = 6), and CNT (*n* = 6) and challenged with 1 μg/mL of LPS for 24 h (LPS), or 1 μg/mL of LPS for 24 h plus 1 mM of ATP for another 15 min (LPS+ATP). Cells were lysed for RNA isolation and expression analysis. The release of IL-1ß was measured in culture supernatants. **(F–H)** Basal expression of *NLRP3, IL1B*, and *IL18* genes were analyzed in untreated MDM using gene-specific Taqman® assays and qPCR. Row target gene expression was normalized by the expression of the *GAPDH* house-keeping gene (ΔCt). Relative expression was calculated as 2^−ΔCt^. Data are expressed as arbitrary units (AU). **(I)** IL-1ß was measured in culture supernatants of treated MDM. **(J–L)** LPS-induced expression of *NLRP3, IL1B*, and *IL18* genes were analyzed in treated MDM using gene-specific Taqman® assays and qPCR. Row target gene expression was normalized by the expression of *GAPDH* house-keeping gene (ΔCt). Modulation of gene expression was calculated as Fold Change (FC = 2 ^−−ΔΔC^) in respect to untreated (UN) cells. Individual data are reported with their mean. The Mann–Whitney test was used to compare two groups. The Kruskall–Wallis test followed by a Bonferroni post-test were used to compare more than two groups. Differences with a *p* < 0.05 were considered statistically significant (*).

In order to better understand the relative contribution of inflammasome activation and IL-1ß release in Mtb infection *in vivo*, we analyzed IL-1ß, IL-18, IFN-α, and TNF levels in the plasma of patients recently diagnosed with active pulmonary TB (TB) and their “household contacts” (CNT) from an endemic TB area (Brazilian State of Amazonas). A group of healthy donors from a non-endemic area (São Paulo, SP, Brazil) was also included. All cytokines, with the exception of IL-18, were up-regulated in TB and CNT individuals with respect to HD (Figures 3A–D). Of note, a significant difference was observed in the plasma level of IL-1ß (Figure 3A) and IFN-α (Figure 3C) between the TB and the CNT groups. The IL-1ß concentration is significantly lower in the TB than in the CNT samples, while the IFN-α level increased in the TB compared to the CNT sample (Figures 3A,C).

These results emphasize that the TB patients presented an imbalance between IL-1ß and IFN-α, as suggested from animal models and *in-vitro* assays (17). These results also show that “household contacts” from endemic areas are characterized by a different plasma cytokine profile compared to TB patients or to the general population from non-endemic TB areas. In part, our findings also correspond with Mayer-Barber et al. (7), even if the unbalance was described specifically for severe TB vs. mild form of the disease in that study.

When we performed a canonical correlation between plasma IL-1ß levels and sputum positivity for Mtb, we observed that the IL-1ß level inversely correlated with the Mtb positive sputum, leading us to clearly separate the CNT and TB groups (Figure 3E). This once more confirms that the production of IL-1ß is an important contributing factor for Mtb infection control, and that this response could be measured systemically.

Next, taking in account the role of NLRP3 in the IL-1ß release by Mtb-infected MDM and the fact that IFN-I is a known inhibitor of NLRP3 (31), we questioned whether the imbalance between IL-1ß and IFN-α, observed in the plasma of TB patients could be related to an impairment of NLRP3 inflammasome.

In an attempt to respond to this tricky question, we first compared the constitutive expression of *NLRP3, CASP1, IL1B*, and *IL18* in MDM from the TB patients, their household contacts (CNT) and heathy donors from a non-endemic region (HD); however, no significant difference resulted among the groups (Figure 3F).

We then treated MDM with 1 μg/mL of LPS for 24 h—with or without the further addition of 1 mM ATP for 15 min—and compared the IL-1ß release among the TB, CNT and HD groups. In cells with normal NLRP3, the addition of ATP significantly increased the LPS-induced release of IL-1ß (24). As expected, ATP boosted IL-1ß release in LPS-treated MDM from HD, and intriguingly also from CNT; however, it did not significantly affect the cytokine release in LPS-treated MDM from TB patients (Figure 3G).

This unresponsiveness of MDM seems to be, at least in part, due to the poor induction of *NLRP3* and *IL1B* expression in TB-treated cells, compared to MDM from HD or CNT (Figure 3H). This condition could be a consequence of high IFN-α production, which possibly prevents NLRP3 inflammasome response (13).

Altogether, these results demonstrate that recently diagnosed TB patients present defective NLRP3 inflammasome and IL-1ß release and, conversely, increased IFN-α production, which can be at least in part responsible for NLRP3 inhibition. Moreover, for the first time to our knowledge, we showed that exposed but healthy subjects (“household contacts”) display an inflammasome response similar to non-exposed healthy donors, allowing us to hypothesize that “Mtb-resistant” individuals may have an IL-1ß production that is better able to counteract IFN-I induction by mycobacteria.

### Inhibition of Cathepsin Activity Abolished IL-1ß and IL-18 Release Suggesting the Contribution of NLRC4 in Inflammasome Activation by Mtb H37Rv

Among typical NLRP3 activation pathways, P2X7-mediaded K+ efflux and lysosomal cathepsins release have been reported as possible mechanisms for an inflammasome activation by mycobacteria (11, 34). To investigate the contribution of these two mechanisms in our model, exogenous ATP (1 mM) or Ca074-Me (10 μg/mL) were added for 15 min after or 1 h before Mtb infection, respectively. As a positive control for NLRP3 activation, MDM were also stimulated with 1 μg/mL of LPS for 24 h with or without 1 mM ATP for another 15 min (24).

**Figure 4:**
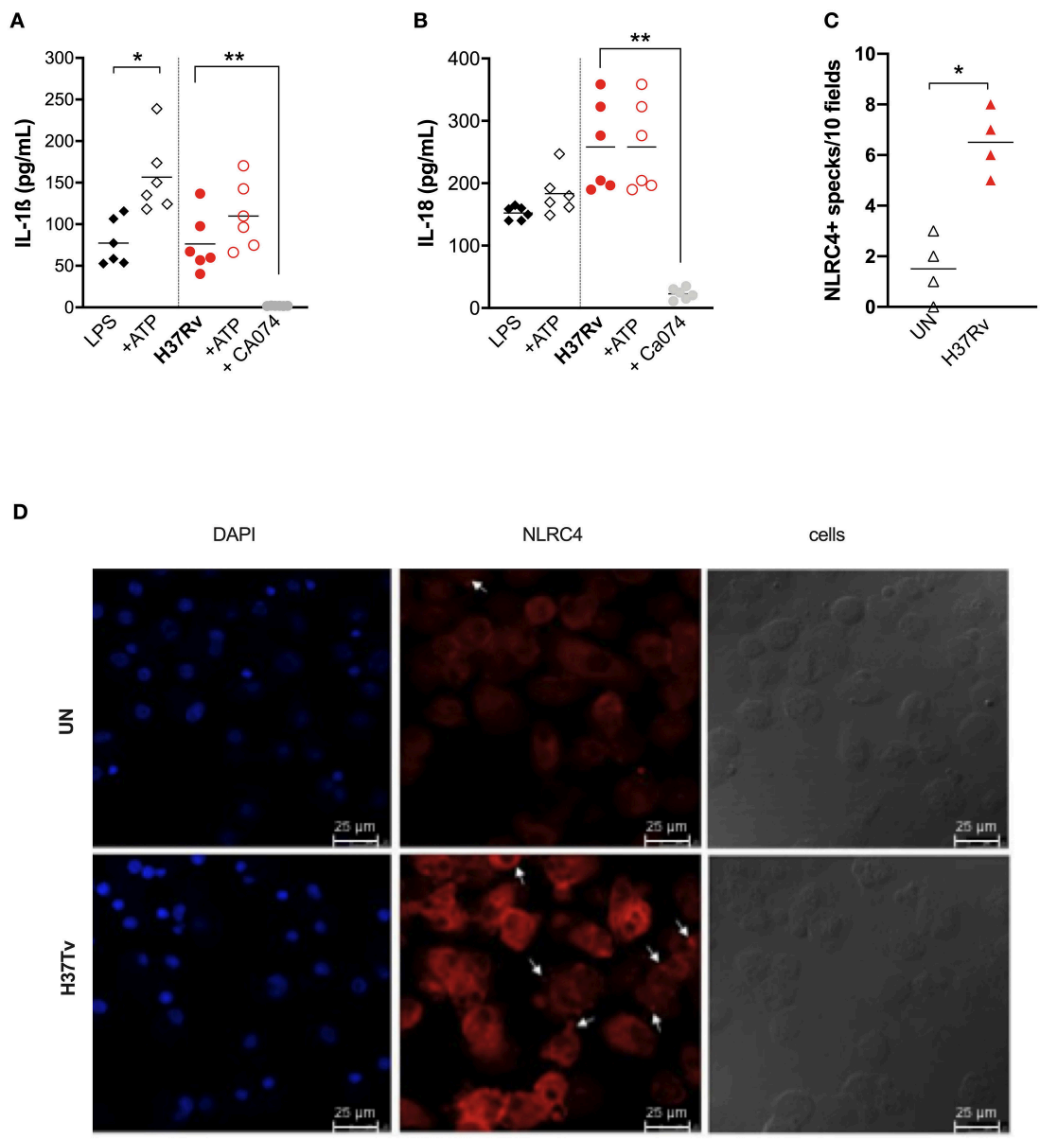
Cathepsins activity is the major mechanism for inflammasome activation by *M. tuberculosis* H37Rv in human MDM, triggering NLRP3 and NLRC4. The healthy donors' monocyte-derived macrophages (MDM; *n* = 6) were treated or not with Ca074-Me (Ca074; 10 μM) before the infection with *M. tuberculosis* H37Rv (MOI: 0.033) for 3 h, then washed and cultured for 24 h. ATP (1 mM) was eventually added for another 15 min in H37Rv-infected MDM. The stimulation of MDM using LPS (1 μg/mL) for 24 h with or without ATP (1 mM) was included as a positive control for NLRP3 inflammasome activation. The release of IL-1ß **(A)** and IL-18 **(B)** was measured in culture supernatants. Healthy donors' monocyte-derived macrophages (MDM; *n* = 4) were cultivated (0.08 × 10^6^) in 16-well chamber slides (*Thermo Fisher Scientific*), challenged with *M. tuberculosis* H37Rv (MOI: 0.033) for 3 h, then washed and cultured for 24 h. Rabbit anti-human NLRC4 and fluorescent secondary antibody (Alexa 647-conjugated goat-anti-rabbit IgG1) were used to label NLRC4. DAPI was used to counterstain the nuclei. NLRC4+ speck formation in untreated (UN) and infected MDM (H37Rv) was analyzed using fluorescence microscopy. The number of NLRPC+ specks was manually counted for each independent experiment **(C)**. A representative experiment was reported. Arrows indicated NLRC4+ specks. **(D)** Individual data are reported with their mean. The Kruskall–Wallis test followed by a Bonferroni post-test were used to compare more than two groups **(A,B)**. The Mann–Whitney test was used to compare treated and untreated groups **(C)**. Differences with a *p* < 0.05 were considered statistically significant (*). **p < 0.001.

Exogenous ATP did not significantly alter inflammasome cytokine production in H37Rv-treated MDM, or in LPS-treated ones (Figures 4A,B), suggesting that, at least in this model, ATP did not amplify mycobacterial stimulation as previously observed in mice (34). On the other hand, the inhibitor of lysosomal cathepsin activity Ca074-Me completely abolished IL-1ß and IL-18 release (96.7 and 97% of inhibition, respectively) (Figures 4A,B), reinforcing previous findings about the role of lysosomal cathepsins release in inflammasome activation by mycobacteria (11).

Taking into account the discrepancy between the only partial reduction in inflammasome activation by MCC-950 (Figures 2A,B), the complete inhibition by Ca074-Me (Figures 4A,B), and the fact that cathepsin release activates another complex receptor, the NLRC4 (35), we hypothesized that this molecule could also be involved in the macrophage response to Mtb. To visualize the NLRC4 participation in inflammasome activation, we once again performed MDM infection with Mtb H37Rv assays and stained the specks using specific antibodies. Samples were observed under a fluorescence microscope. A significant increase in the number of NLRC4+ specks was observed in Mtb-infected MDM compared to untreated cells (Figures 4C,D).

These findings show that lysosomal cathepsins represent the main pathway for NLRP3 triggering by H37Rv, and maybe also for NLRC4, a receptor which is poorly investigated in TB and pulmonary diseases.

### Genetic Variants in *NLRP3* and *NLRC4* Genes Distinctively Affect the Host Response to *M. tuberculosis* Infection and Outcome

Once confirming the central role exerted by NLRP3 in the inflammasome activation and host response against virulent Mtb, we next investigated the contribution of the TB protective *NLRP3* variant rs10754558 (18) on this response.

**Figure 5:**
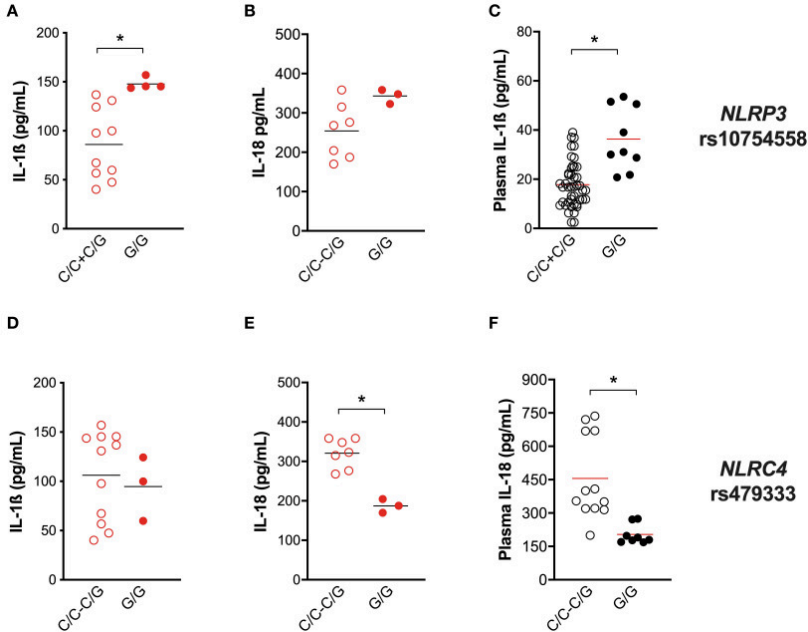
Genetic variants in *NLRP3* and *NLRC4* genes affect host response to *M. tuberculosis*. The healthy donors' monocyte-derived macrophages (MDM; *n* = 14) were treated with *M. tuberculosis* H37Rv (MOI: 0.033) for 3 h, then washed and cultured for 24 h. The IL-1ß and IL-18 level was measured in culture supernatants at the end of experiment (*n* = 14 and 8, respectively), and in the plasma of TB patients (*n* = 27 and 20, respectively). *NLRP3* rs10754558 and *NLRC4* rs479333 SNVs were genotyped in the genomic DNA of healthy donors and TB patients. IL-1ß and IL-18 release data were then grouped by individual *NLRP3* rs10754558 genotype into homozygotes for the minor G allele (G/G) and the others (C/C+C/G), according to a recessive model of inheritance for the minor allele **(A–C)**; or by individual *NLRC4* rs479333 genotype into homozygotes for the minor C allele (C/C) and the others (G/G+G/C) according to a recessive model of inheritance for the minor allele **(D–F)**. Individual data are reported with their mean. The Mann–Whitney test was used to compare the groups. Differences with a *p* < 0.05 were considered statistically significant (*).

For this purpose, IL-1ß and IL-18 release from Mtb-challenged HD MDM was compared in carriers and non-carriers of the variant. As expected, individuals carrying the gain-of-function rs10754558 C>G variant in homozygosis (G/G) produced more IL-1ß when stimulated with the Mtb H37Rv than non-carriers (Figure 5A), confirming that NLRP3 is a crucial inflammasome sensor for Mtb. Moreover, in the presence of high levels of NLRP3 (28), macrophages respond to Mtb processing a larger amount of IL-1ß. Intriguingly, the IL-18 release in Mtb-treated HD MDM was not significantly affected by this SNV (Figure 5B). Then we performed the genotype-guided analysis with plasma cytokines levels in TB patients TB patients carrying rs10754558 G/G displayed an increased level of IL-1ß compared to non-carriers (Figure 5C), emphasizing that the effect of the NLRP3 gain-of-function variant against Mtb is detectable also *in vivo*. The SNV did not affect the inflammasome-dependent cytokine TNF (Supplementary File 2A).

In our previous genotyping analysis (18), we did not include the *NLRC4* gene. At that time, genetic association studies did not refer to *NLRC4* as an important locus for Mtb infection. Only recently, it was reported that *NLRC4* SNVs may affect the outcome of TB in chronically infected HIV patients (36). As our *in vitro* results indicate the participation of NLRC4 in inflammasome activation by virulent Mtb (Figure 4), we selected a known functional variant in *NLRC4* (rs479333 G>C; found in 45% of the general population) and performed a genotype-guided analysis. The loss-of-function rs479333 variant did not affect IL-1ß in our *in-vitro* model of infection (Figure 5D), but was associated with a lower level of IL-18 both *in vitro* (Figure 5E) and *in vivo* (TB patients' plasma) (Figure 5F). The SNV did not affect the inflammasome-independent cytokine TNF (Supplementary File 2B).

These findings functionally demonstrate the effect of *NLRP3* rs10754558 SNV on TB protection (18), emphasizing the key role of NLRP3 in the first contact host/mycobacteria. Moreover, they suggest that *NLRC4* genetics may also affect Mtb/host interplay, even if in a different way.

Trying to assess whether *NLRC4 r*s479333 can represent a susceptibility factor for TB, we performed an association study in a case/control cohort of TB. A multivariate analysis was first executed comparing *NLRC4 r*s479333 and *NLRP3* rs10754558 distribution between TB patients and healthy donors. Then, to detect the possible effect on clinical presentation, we compared SNVs distribution in TB patients with pulmonary and with extra-pulmonary forms. The results are presented in Table 3.

**Table 3:** Association results for SNVs in inflammasome-encoding genes in the TB cohort from the Brazilian State of Amazonas.

**Gene SNV ID**	**Effect**	**Genotypes**	**HD (*n* = 288)**	**TB (*n* = 352)**	**p_**adj**_ OR_**adj**_ (95%CI)**	**PTB (*n* = 288)**	**ETB (*n* = 64)**	**p_**adj**_ OR_**adj**_ (95%CI)**
*NLRP3*rs10754558	GoF	C/C	0.52	0.65	**0.003** 0.16 (1.02–1.29)	0.68	0.62	0.475 0.96 (0.64–3.56)
		C/G	0.35	0.31		0.27	0.34	
		G/G	0.13	0.04		0.05	0.04	
*NLRC4*rs479333	LoF	G/G	0.66	0.49	0.658 0.78 (0.25–2.43)	0.39	0.60	**0.0006** 0.20 (1.92–13.16)
		C/G	0.20	0.31		0.39	0.23	
		C/C	0.14	0.20		0.22	0.17	

*Gene symbol, variant identification number (SNV ID), genotypes frequencies for patients with active pulmonary TB (PTB) extra-pulmonary (ETB) and healthy donors (HD), GLM p-value (p), odds ratio (OR) and 95% confidence interval (CI) are reported adjusted for sex and age (p_adj_, OR_adj_). Statistically significant results after Bonferroni correction are indicated in bold characters*.

As we have previously shown (18), *NLRP3* rs10754558 represents a protective factor against TB susceptibility, being more frequent in controls (G/G: 0.13) than in patients (0.04). On the other side, and for the first time to our knowledge, we observed that the loss-of-function variants in *NLRC4* (rs479333), which did not affect susceptibility to TB *per se* (p_adj_ = 0.658), results significantly less frequent in patients with the more severe form of infection (extra-pulmonary TB) compared to pulmonary TB, according to a dominant model of inheritance for the minor C allele (G/C+C/C: 0.40 vs. 0.61) (Table 3).

This genetic approach was not only useful for supporting the previously published association of the *NLRP3* rs10754558 variant with Mtb infection (18), but also for revealing that *NLRC4* could be another genetic factor important for the outcome of infection. It is interesting to emphasize that the two studied SNVs (rs10754558 and rs479333) principally affect one or the other inflammasome cytokine in Mtb-infected MDM (IL-1ß and IL-18, respectively), suggesting the distinct contribution of the mediators in TB.

### Mtb Strains of Different Virulence and the Host's Genetic Background Shape NLRP3 Inflammasome Response to Mycobacteria

As the development of active TB is the complex outcome of several factors, including genetics, environment and pathogen biology (3), the next step was to evaluate the impact of Mtb phenotype on the inflammasome activation together with the host's genetic background. For this purpose, and considering the emergence of TB patients with a severe presentation caused by hyper-virulent strains (37, 38), MDM were challenged with different strains of mycobacteria: BCG and MP287/03 (MP) non-virulent and hyper-virulent strains from *M bovis* respectively; and the hyper-virulent strain Beijing-1471 (Bj) from *M. tuberculosis*. IL-1ß and IL-18 release was compared with the common virulent strain H37Rv, also from *M. tuberculosis*.

**Figure 6:**
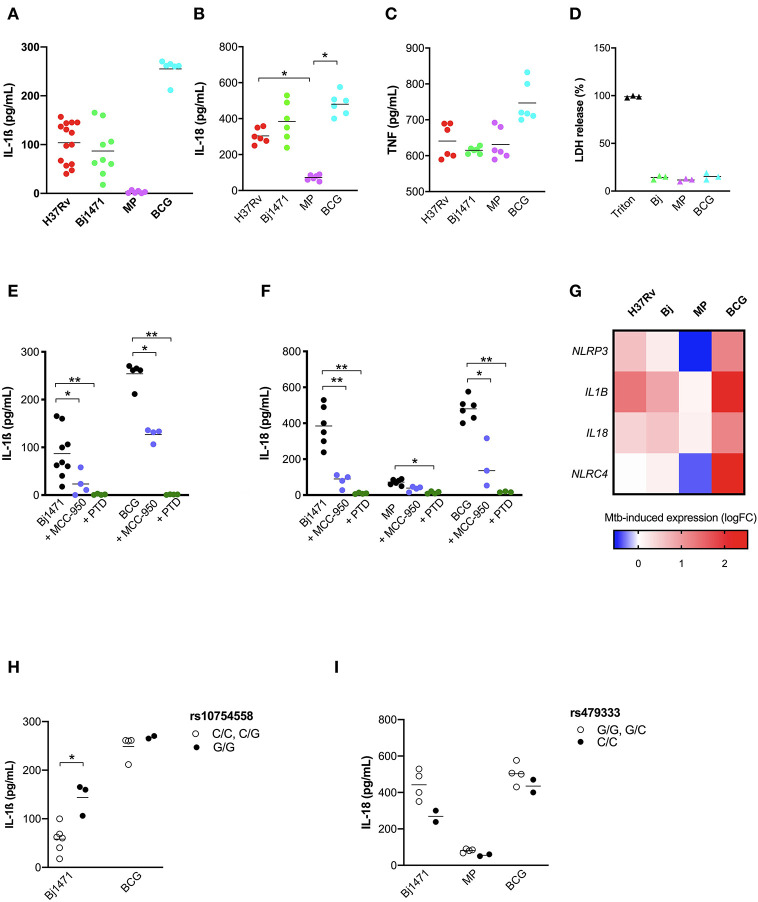
Mtb strains of different virulence and host genetic background shaped the inflammasome response to mycobacteria. The healthy donors' monocyte-derived macrophages (MDM) were treated with 10 μM MCC-950 or 10 μM parthenolide (PTD) 1 h before infection with *M. tuberculosis* (MOI: 0.033) H37Rv (*n* = 14), Beijin-1470 (Bj-1470; *n* = 9), MP287/03 (MP; *n* = 6) and *Bacillus Calmette Guerin* (BCG; *n* = 6) for 3 h, then washed and cultured for 24 h. The release of IL-1ß **(A)**, IL-18 **(B)**, TNF **(C)**, and LDH **(D)** was measured in culture supernatants. Dosage for IL-1ß was executed for all samples, while for IL-18 and TNF in six samples, for LDH in three sample. Cytokines and LDH release were compared among different Mtb strains. Cells were lysed for RNA isolation and gene expression analysis. *NLRP3* rs10754558 and *NLRC4* rs479333 SNVs were genotyped in the genomic DNA of healthy donors. The effect of MCC-950 and PTD on inflammasome activation in infected MDM was analyzed by the meaning of IL-1ß **(E)** and IL-18 **(F)** release. Mtb-induced expression of *NLRP3, NLRC4, IL1B*, and *IL18* genes in H37Rv-, Bj-1470-, MP- and BCG-infected MDM was calculated as 2^−ΔΔCt^ (fold-change, FC) and reported as log_2_FC in a heat-map graph **(G)**. IL-1ß release data were grouped by individual *NLRP3* rs10754558 genotype into homozygotes for the minor G allele (G/G) and the others (C/C+C/G), according to a recessive model of inheritance for the minor allele **(H)**. IL-18 release data were grouped by *NLRC4* rs479333 genotype into homozygotes for the minor C allele (C/C) and the others (G/G+G/C) according to a recessive model of inheritance for the minor allele **(I)**. Individual data are reported with their mean. The Kruskall-Wallis test followed by a Bonferroni post-test were used to compare groups **(A–F)**. The Mann-Whitney test was used to compare two groups **(H,I)**. Differences with a *p* < 0.05 were considered statistically significant (*). **p < 0.001.

H37Rv and Bj strains induced a similar amount of IL-1ß, but higher than MP, providing to be a poor IL-1ß producer. BCG was the major inductor of IL-1ß, leading to about a 3-fold increase in cytokine compared to H37Rv or Bj (Figure 6A). This difference among BCG, H37Rv, and Bj was not as important in the case of IL-18 production, even if BCG tends to induce a higher amount of cytokine, especially when compared to H37Rv (Figure 6B). MP was able to induce significant IL-18 release compared to untreated MDM, even if in a lower amount compared to other strains (Figure 6B). Of note, the production of TNF was similar in all conditions (Figure 6C), as well as the LDH release (Figure 6D).

To depict the relative contribution of NLRP3 in sensing distinct Mtb strains, we performed assays in the presence of the NLRP3 specific inhibitor MCC-950 or the general inhibitor PTD. MCC-950 inhibited about half of IL-1ß and IL-18 production in H37Rv-, Bj-, and BCG-treated -MDM. Due to the low production of IL-18 induced by MP the inhibition rate observed for MCC-950 was not statistically significant (*p* = 0.08) (Figures 6E,F). As expected, PTD completely abolished IL-1ß and IL-18 production in all the conditions (Figures 6E,F).

The pattern of IL-1ß and IL-18 release by MDM was distinct depending on the Mtb strain, and these differences may be related to the different ability of Mtb strains to induce inflammasome genes expression (Figure 6G). In terms of cytokine production, BCG promotes the highest expression of the 4 analyzed genes *(NLRP3, NLRC4, IL1B*, and *IL18*), and MP the lowest.

Finally, to assess the contribution of host genetics on the inflammasome activation toward different Mtb strains, we performed genotype-guided assays for the two functional variants *NLRP3 r*s10754558, and *NLRC4* rs479333. Despite the limited number of tested samples (*n* = 12), MDM from donors carrying the gain-of-function *NLRP3* variant (G/G) responded to Beijin-1470 and BCG strains, producing a higher level of IL-1ß compared to MDM from non-carriers (C/C+C/G) (Figure 6H).

At the same time, and contrary to what was observed for H37Rv-treated MDM, the loss-of-function variant *NLRC4* rs479333 did not significantly affect IL-18 release in MDM challenged with BCG. Beijin-1470 or MP287/03 (Figure 6I).

This last set of experiments demonstrated that different strains of Mtb display a unique capacity to induce inflammasome activation at a rather physiologic condition (MOI: 0.033) with virulence (non-virulent more than virulent) playing a greater role than the type of bacteria (*M.bovis* BCG more than *M.bovi*s MP287/03; *M. tuberculosis* H37Rv similar to *M. tuberculosis* Beijin-1470). Moreover, we showed that host genetics may affect the inflamamsome activation rate toward different Mtb strains, especially for *NLRP3* rs10754558 SNV—once again corroborating the important role of inflammasome genetics in individual responses to mycobacteria.

## Discussion

Extensive efforts have been made to better understand the mechanisms involved in Mtb/host interactions and how this complex interplay may determine resistance vs. susceptibility to Mtb and the development of TB. Innate immunity and first-line immune responses, including inflammasome and related cytokines, have recently been taken into account as important determinants in TB outcomes. Notwithstanding, our knowledge of the mechanisms underlying progression of Mtb infections and the development of the active disease in humans is not yet fully understood, in part due to the lack of appropriate models of study, and also due to the difficulty of interpreting complex genetics, immunologic, and microbiologic interactions.

Starting from our previous genetic data (18), we designed a study for the contemporary evaluation of how inflammasome genetics and Mtb phenotype affect inflammasome activation in primary human monocytes-derived macrophages, in turn contributing to individual responses to Mtb and/or to Mtb-susceptibility vs. resistance establishment.

Our findings show that the common virulent strain H37Rv induces NLRP3 inflammasome activation (but not pyroptosis) in human macrophages and that IL-1ß is mandatory for bacterial containment (Figures 1, 2). These findings corroborate previous studies in murine and human macrophages (10–12, 17, 31, 32); however, it is important to mention that in those studies, the MOI level was very high (10 or higher), while we decided to infect cells with an MOI level that was as physiologic as possible (0.033). This protective response can be modulated by host genetics, as macrophages from individuals carrying the gain-of-function rs10754558 in *NLRP3* produce significantly more IL-1ß than non-carriers (Figure 5A), as well as by the type of Mtb strain, as non-virulent BCG strains trigger a large amount of cytokine while the hyper-virulent MP287/03 none at all (Figure 6A). These data support the protective effect observed for *NLRP3* rs10754558 against the development of active pulmonary TB (18).

Trying to better understand how the host/mycobacteria interplay acts *in vivo*, we analyzed the plasma levels of inflammasome cytokines, IFN-α and TNF in a group of recently diagnosed TB patients (before treatment) and their “household contacts”—representing a group of exposed but healthy subjects. As expected, responder individuals (“household contacts”) presented a higher level of IL-1ß, suggesting that their inflammasome is functioning well (Figure 3). On the contrary, susceptible individuals (TB patients) were characterized by an imbalance between IL-1ß and IFN-α, a known inhibitor of NLRP3 inflammasome (13). This concept of fine tuning between innate immune mediators was previously proposed by Mayer-Barber et al. (7), referring to the severity of the TB presentation; however, our study demonstrates that a “good” inflammasome activation is determinant for Mtb resistance in exposed population such as endemic TB areas. Intriguingly, TB patients also appeared to be less prone to an efficient response against PAMPs/DAMPs, as suggested by impaired NLRP3 activation by common stimuli (Figure 3). A similar impairment of NLRP3 was first reported in the dendritic cells of HIV patients (39), which are also characterized by elevated IFN-I levels, emphasizing a common inhibitory mechanism mediated by the antiviral mediator. Wassermann and colleagues aptly demonstrated that Mtb evolved some strategies to by-pass inflammasome activation, one being to activate pathways leading to IFN production (17).

According to Amaral et al. (11), H37Rv also activates the inflammasome in human macrophages through lysosomal damage and cathepsins release, as demonstrated by the complete inhibition of cytokine release by Ca074-Me (Figures 4A,B). However, the discrepancy between the dramatic effect of Ca074-Me and only partial inhibition obtained with the NLRP3 specific inhibitor MCC-950 (23) led us to hypothesize the contribution of another pathway for inflammasome activation. Besides NLRP3, other inflammasome receptors, such as the NLRC4, have been shown to contribute to the host's response to intracellular bacteria (9). Differently from NLRP3, NLRC4 can either directly recognizes bacterial patterns, such as flagellin (40), or be activated by lysosomal cathepsins release, at least in mice (35). The role of NLRC4 in TB remains elusive because it has been poorly investigated in TB and pulmonary diseases. Microscopic visualization of inflammasome aggregates (“specks”), allowed us to observe NLRC4 in the mounted complex, suggesting that it can contribute to macrophages response against Mtb; however, further investigation will be needed to better depict the role of this molecule in TB pathogenesis.

Through a genetic approach and genotype-guided assay, for the first time to our knowledge, we found a significant association between NLRC4 and the outcome of TB in an endemic area. The loss-of-function variant rs479333 in the *NLRC4* gene (minor allele frequency, 49%; from ensembl.org) was less frequent in patients with extra-pulmonary TB than in patients with pulmonary disease (Table 3). This SNV, previously associated to reduced *NLRC4* transcription (29) and diminished plasma level of IL-18 (30), was associated with a lower release of IL-18 in H37Rv-challenged MDM as well as the plasma level of the cytokine (Figures 5E,F), reinforcing its loss-of-function effect at both local and systemic levels.

We would like to emphasize that our data reveal two situations in Mtb/host interplay: one characterized by the preferential NLRP3/IL-1ß axis and another by NLRC4/IL18 axis. The first exerts a central role in first host/pathogen contact and in mycobacterial containment while the other is apparently more important in the outcome of Mtb infection. As we know, IL-1ß and IL-18 assume distinct roles within immune response. While IL-1ß is mainly involved in endothelium and phagocytes activation, IL-18 is involved in epithelium homeostasis and CD4+ T lymphocytes polarization toward Th1 IFN-γ-producer cells.

Considering the importance of IFN-γ-producer cells in typical TB granuloma formation, we therefore hypothesize that individuals with a defective NLRC4/IL18 axis are not able to effectively contain mycobacteria within granulomas, leading to eventual extra-pulmonary dissemination. Accordingly, at least in HIV-infected patients, the expression of *NLRC4* significantly increased in the blood of individuals with a more severe form of TB (41). Moreover, it is interesting to remember that loss-of-function SNVs in *NLRC4* have been found in HIV patents with severe TB (36) and in patients with cystic fibrosis affected by severe lung infections (42), reinforcing the important and poorly investigated role of NLRC4 in lung immunity and pulmonary diseases.

Mtb strains induce inflammasome activation in a “virulence-dependent” way. The non-virulent strain BCG promotes a dramatic inflammasome activation compared to common virulent (H37R) and rare hyper-virulent (Beijing 1471, MP287/03) strains, suggesting that Mtb virulence evolved together with some mechanism of inflammasome escape (17), and corroborating once more the importance of inflammasome activation in the immune response against Mtb.

Of note, the pattern of IL-1ß and IL-18 release is not identical in Mtb-challenged MDM (Figure 6), being IL-18 levels higher than IL-1ß for all tested strains. Moreover, IL-1ß production appears to be more virulence-dependent than IL-18. These findings suggest that Mtb could activate several and distinct inflammasome pathways (i.e., NLRP3/IL-1ß or NLRC4/IL-18 axis) compared to a unique PAMP, such as LPS, and these pathways could alternatively process IL-1ß or IL-18, as previously proposed (8, 9). The different level of inflammasome activation in response to distinct Mtb strains has also been observed in the murine model of infection, showing that MP287/03 is a poor inducer of IL-1ß both *in vivo* and *in vitro* compared to H37Rv or Beijing; IL-18 was not, however, evaluated in these models (34).

Masters et al. (43) proposed that the difference in the level of inflammasome activation by distinct Mtb strains is due to the virulence factor *Zmp1*. Interestingly, this group has also noted that MP287/03, which does not express *Zmp1*, activates inflammasome through NLRC4. In our assays, MP287/03 induced IL-18 but not IL-1ß. However, this Mtb strain is not able to trigger NLRC4, nor NLRP3 expression (Figure 6). Consequently, we cannot conclude whether Mtb strains which preferentially induce IL-18 do so through NLRC4. Nevertheless, taking into account the long co-evolution period of Mtb and humans, it would not be surprising that several inflammasome sensors could contribute to mycobacterial defense, as it was demonstrated for NLRP3 and AIM2 (17).

In conclusion, for the first time, our findings show the intricate interaction among inflammasome genetics, Mtb virulence and the resultant inflammasome activation profile at the local (*in vitro* model) as well at the systemic (plasma) level. According to our hypothesis, during the first host/pathogen contact, the Mtb activates a strong inflammasome response in term of IL-1ß as well as IL-18, especially for non-virulent strain BCG and the common virulent one, H37Rv. This response is mediated by the NLRP3/IL-1ß axis in a cathepsin-dependent way, and the gain-of-function variant in NLRP3 contributes to the elevated production of IL-1ß, which in turn is specifically responsible for bacterial containment.

**Figure 7:**
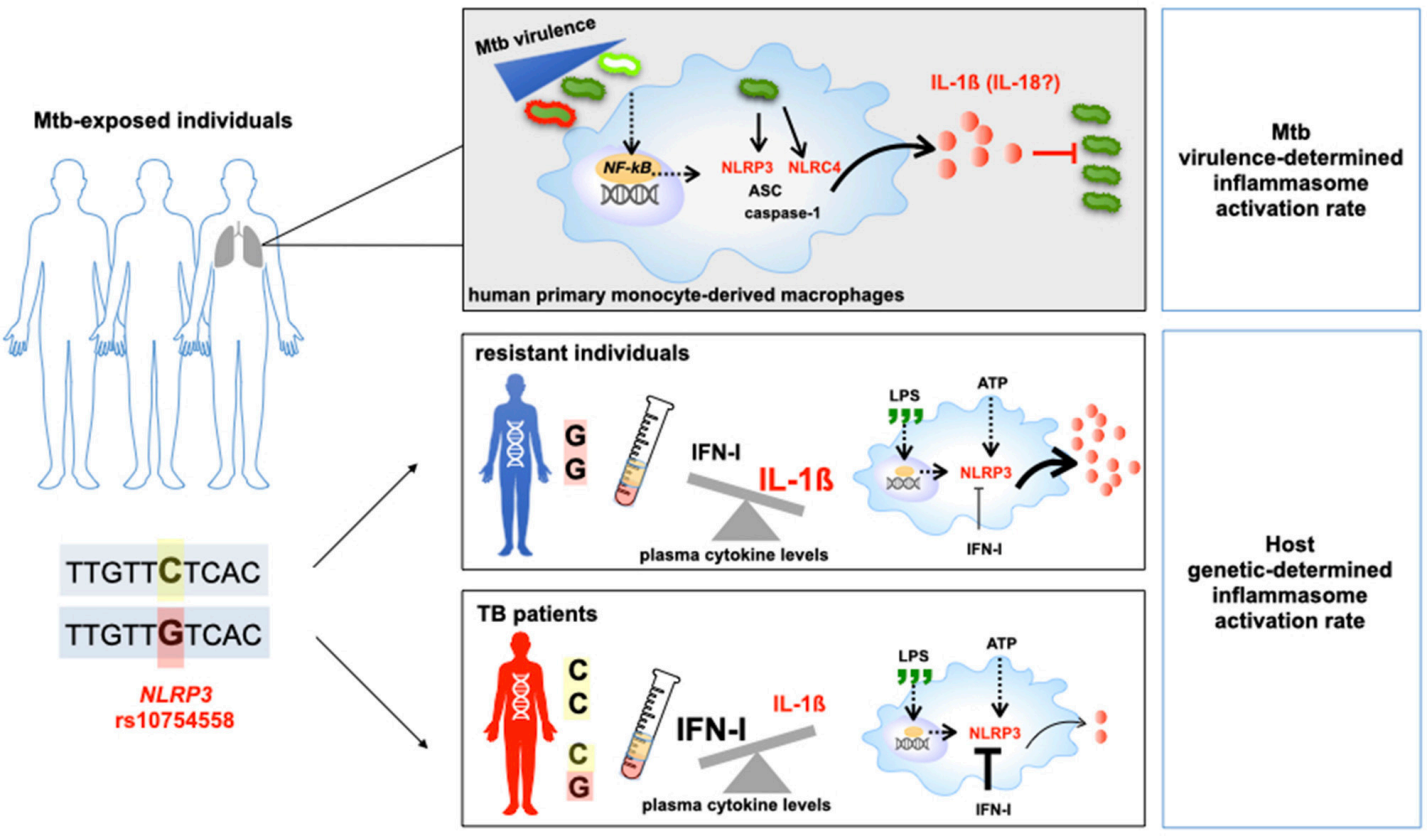
Host genetic background and *M.tuberculosis* (Mtb) determine the outcome of TB. Schematic representation of the main results of this study.

*In-vitro* assays and genetic association data support the involvement of the NLRC4/IL18 pathway in individual responses to mycobacteria and determine the outcome of an Mtb infection as well as TB severity. These original findings highlight that the same pathogen could activate inflammasome-dependent cleavages of cytokine pro-forms based on events that are not yet fully understood, such as a bacterial strain (i.e., MP287/03), distinct steps of the disease (i.e., first host/pathogen contact, or response to the pathogen in an individual with an established infection), or even based on an individual's genetic background (Figure 7).

## Data Availability Statement

The raw data supporting the conclusions of this article will be made available by the authors, without undue reservation, to any qualified researcher.

## Ethics Statement

This study was approved by the Human Research Ethics Committee of the Institute of Biomedical Sciences (ICB) of the University of São Paulo (USP) (CEPSH reference number: 51780715.8.0000.5467) and the Institute of Biological Sciences (ICB) of the Federal University of Amazonas (UFAM) (reference number: 57978916.3.0000.5020). All human participants provided written informed consent and all clinical investigations were conducted according to the principles expressed in the Declaration of Helsinki. The patients/participants provided their written informed consent to participate in this study.

## Author Contributions

DS and AP: conceived, designed experiments, wrote the article, and conducted statistical analyse. AS and MO: selected and collected TB patients and household contacts. MO: performed microbiologic analysis. DS and CB: performed MDM experiments. EA and CB: standardized *Mycobacterial* culture and infection. DS and VL: performed the immunofluorescence experiments. ER: performed flow-cytometry experiments. FF and JS: executed ELISA, performed RNA isolation, and gene expression analyse. DS, AP, FL, MO, AS, and ML: discussed the results. All authors contributed to the article and approved the submitted version.

## Conflict of Interest

The authors declare that the research was conducted in the absence of any commercial or financial relationships that could be construed as a potential conflict of interest.
