# Supplementary material for: Combining Host Genetics and Functional Analysis to Depict Inflammasome Contribution in Tuberculosis Susceptibility and Outcome in Endemic Areas
Source: Front Immunol. 2020 Oct 21;11:550624. doi: 10.3389/fimmu.2020.550624 (PMC7609898; doi:10.3389/fimmu.2020.550624)

Supplementary Material

Supplementary File 1. Monocytes-to-MDM differentiation analysis. Representative gating strategy. Singlet cells were gated along the diagonal when forward scatter height (FSC-H) versus forward scatter area (FSC-A) was plotted **(A).** Cells were selected according to size and granularity **(B)** followed by live/dead discrimination based on Live or Dead negative cells (live) **(C).** Finally, cells double positive for CD14 and CD68 were selected **(D).**

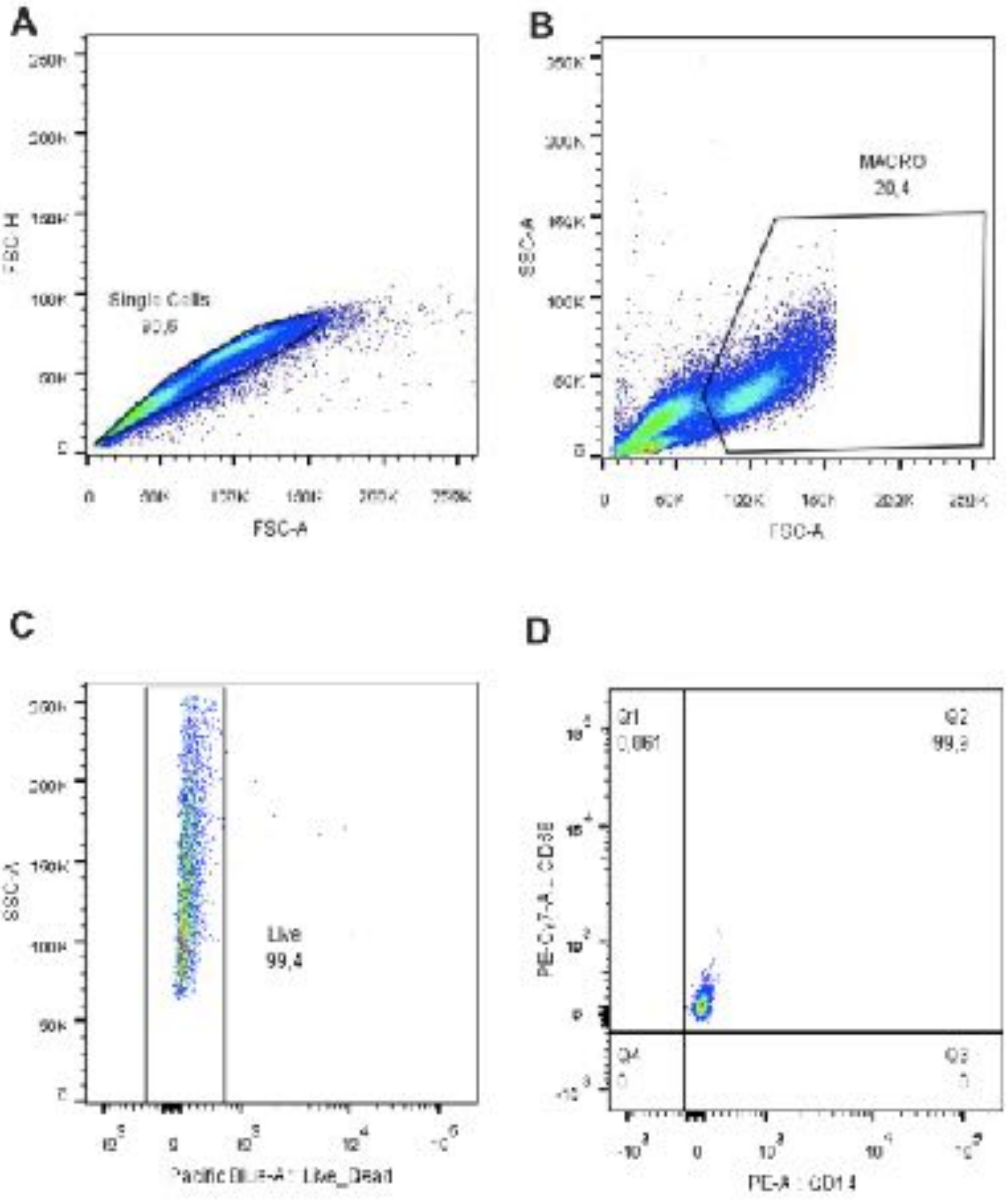

Supplementary File 2. Genetic variants in *NLRP3* and *NLRC4* genes did not affect TNF secretion by Mtb-infected MDM. Healthy donors monocyte-derived macrophages (MDM; n=14) were treated with *M.tuberculosis* H37Rv (MOI: 0.033) for 3 hours, then washed and cultured for 24 hours. TNF secretion was measured in culture supernatants at the end of experiment. *NLRP3* rs10754558 and *NLRC4* rs479333 SNPs were genotyped in the genomic DNA of healthy donors. TNF release data were then grouped according to individual *NLRP3* rs10754558 genotype into homozygotes for the minor G allele (G/G) and the others (C/C+C/G), according to a recessive model of inheritance for the minor allele (**A**); or according to *NLRC4* rs479333 genotype into homozygotes for the minor G allele (G/G) and the others (C/C+C/G), according to a recessive model of inheritance for the minor allele **(B)**. Mann-Whitney test was used to compare the groups. Differences with a p<0.05 were considered statistically significant (*).

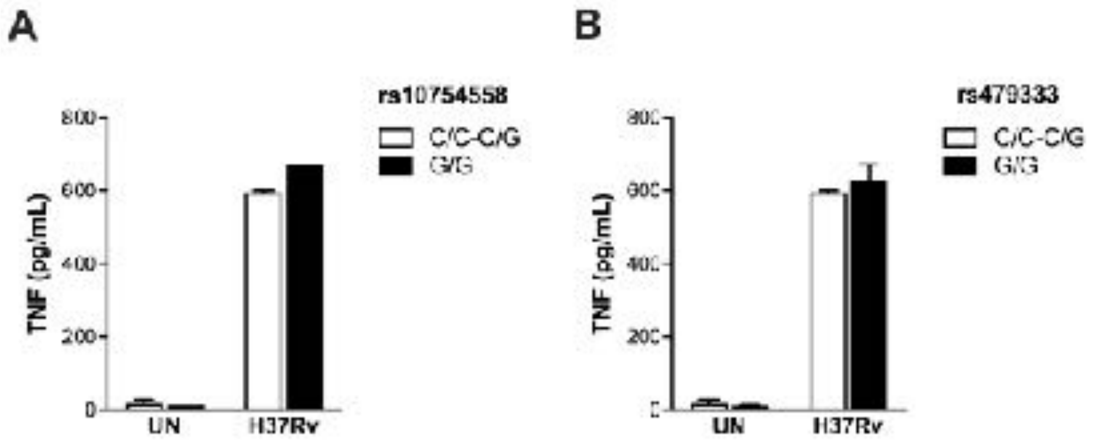

Supplement: Supplementary file 1 [file Data_Sheet_1.PDF]
